# A SHA-256 Hybrid-Redundancy Hardware Architecture for Detecting and Correcting Errors

**DOI:** 10.3390/s22135028

**Published:** 2022-07-03

**Authors:** Ignacio Algredo-Badillo, Miguel Morales-Sandoval, Alejandro Medina-Santiago, Carlos Arturo Hernández-Gracidas, Mariana Lobato-Baez, Luis Alberto Morales-Rosales

**Affiliations:** 1 Department of Computer Science, CONACYT-National Institute for Astrophysics, Optics and Electronics, Puebla 72840, Mexico; algredobadillo@inaoep.mx (I.A.-B.); amedina@inaoep.mx (A.M.-S.); 2 Center for Research and Advanced Studies of the IPN-CINVESTAV, Unidad Tamaulipas, Ciudad Victoria 87130, Mexico; miguel.morales@cinvestav.mx; 3 Facultad de Ciencias Físico Matemáticas, CONACYT-Benemérita Universidad Autónoma de Puebla, Puebla 72570, Mexico; cahernandezgr@conacyt.mx; 4 Instituto Tecnológico Superior de Libres, Libres, Puebla 73780, Mexico; mariana.lobato@upaep.edu.mx; 5 Facultad de Ingeniería Civil, CONACYT-Universidad Michoacana de San Nicolás de Hidalgo, Morelia 58030, Mexico

**Keywords:** fault detection, hardware redundancy, hardware architecture, pipeline architecture, SHA-256, time redundancy

## Abstract

In emergent technologies, data integrity is critical for message-passing communications, where security measures and validations must be considered to prevent the entrance of invalid data, detect errors in transmissions, and prevent data loss. The SHA-256 algorithm is used to tackle these requirements. Current hardware architecture works present issues regarding real-time balance among processing, efficiency and cost, because some of them introduce significant critical paths. Besides, the SHA-256 algorithm itself considers no verification mechanisms for internal calculations and failure prevention. Hardware implementations can be affected by diverse problems, ranging from physical phenomena to interference or faults inherent to data spectra. Previous works have mainly addressed this problem through three kinds of redundancy: information, hardware, or time. To the best of our knowledge, pipelining has not been previously used to perform different hash calculations with a redundancy topic. Therefore, in this work, we present a novel hybrid architecture, implemented on a 3-stage pipeline structure, which is traditionally used to improve performance by simultaneously processing several blocks; instead, we propose using a pipeline technique for implementing hardware and time redundancies, analyzing hardware resources and performance to balance the critical path. We have improved performance at a certain clock speed, defining a data flow transformation in several sequential phases. Our architecture reported a throughput of 441.72 Mbps and 2255 LUTs, and presented an efficiency of 195.8 Kbps/LUT.

## 1. Introduction

In the short term, the availability of digital devices, along with their inherent need for interconnectivity, will raise several challenges in security, power resources, hardware, and software saturation, among others. For modern contexts of emergent technologies, such as cyber-physical systems, vehicular communication networks and Industry 4.0, assuring data integrity, confidentiality and authentication for message-passing communications is a critical requirement, where cryptographic solutions are among the most important alternatives.

Security is a constant and real concern, even more so if the application environments bring related issues along, such as failures, transfer errors, viruses, malware, compromised hardware, or physical damage to devices. These issues are also related to attackers, who may modify, replace, or damage data, causing accidents, financial losses, or different kinds of problems. Besides, faults are inherent in some hardware architectures, or they can be generated by their environment, being classified as transient or permanent. On the one hand, transient faults are considered as single-event upsets(SEU) when there is a change of logic state or multiple transient faults are triggered by several SEU. On the other hand, permanent faults cause defects in system behavior and produce halts or wrong results [1].

Nowadays, security services for integrity, confidentiality, and authentication must consider faults, which are more critical if cryptographic algorithms are taken into account, due to their behavior focused on several iterations and processing all message data. In this case, the type of cryptographic algorithms known as hash functions (because they generate a hash from some message constituted by bits, bytes, words, images, videos, text pages, etc.) present high diffusion of all the bits in processing time. The desirable characteristics of these algorithms can be greatly affected by faults, since changing a single bit will produce a totally different hash.

Moreover, providing an autonomous-decision mechanism to determine if the data integrity calculation has been appropriately carried out, to assure system consistency, is a key design element of digital communication systems [2].

In this sense, faults and errors caused by hardware noise or data transmission alterations, generated either by the channel quality or by external attacks, should be detected quickly and efficiently for a fault-detection module to be considered suitable for real-time applications. As an important remark, when the input and output data differ, it means an error has occurred. For instance, in message-passing communications, the presence of noise can affect the channel during transmission of bits, which can be contaminated by errors. Besides, errors can appear from a miscalculation in the microprocessors or adverse scenarios such as attackers or physical damage. An example of this is when a bit with value 0 is changed to 1, or vice versa, in the payload.

Secure Hash Algorithm (SHA) functions offer integrity, although they can be employed to provide authentication. Besides, the algorithm does not include verification mechanisms for its inside operations and future fails at the system level.

Noise and possible failures in the system are critical within the design of hardware implementations [3,4,5]. For instance, several works have demonstrated that noise and interference affect the operation of FPGAs, such as it happens in the presence of magnetic noise in the white noise region [6]. When the voltage supply is unstable, this can negatively affect the immunity of the I/O buffer by about 16.8 dB, according to Toan et al. [7]. Therefore, we considered developing hardware modules capable of assuring integrity, security and data quality with a system-level fault detection mechanism.

Traditional electronic systems designed for fault tolerance are based on redundancy [8,9]. Applications where failure is critical use redundant pieces of hardware and software. In such systems, time redundancy, information redundancy, and hardware redundancy can also be considered.

Best practices in data integrity consider validation to prevent the entrance of invalid data, perform error detection in transmissions, and execute security measures against data modifications or for loss prevention [8]. SHA-2 is a broad family of solutions to assure data integrity, and the standard [9] defines several configurations for these cryptographic hash functions, generating hashes with 256, 384, and 512 bit sizes. The SHA-256 algorithm is widely used to balance security and fast implementation for contexts that require a real-time response based on hardware. Current hardware implementations of SHA-2 are focused on satisfying those characteristics, but there are issues regarding real-time balance among processing, efficiency, and cost, so diverse researches and analysis should be pursued.

Cryptographic algorithms can be used for different security related tasks such as confidentiality, authentication, integrity, and non-repudiation. Among the most important algorithms, we have hash functions used in the blockchain, digital signature, digital certificates, and other applications. SHA-2, designed by the National Security Agency (NSA), is a set of cryptographic hash functions, which includes important changes with respect to its predecessor (SHA-1). It consists of six hash functions and digests of 224, 256, 384, or 512 bits. While SHA-256 is a hash function with 32-bit words, SHA-512 processes 64-bit words instead. The two variations have identical structures and differ only in the amounts of displacement, additive constants, and rounds. On the other hand, we can consider SHA-224 and SHA-384 as truncated versions of SHA-256 and SHA-512, respectively, which are calculated using different initial values. Other truncated versions of SHA-512, also with different initial values, are SHA-512/224 and SHA-512/256.

In this work, we identify an open problem: How to ensure a fast and reliable fault detection architecture even in adverse situations, which can be integrated into several environments without representing an overload to the system? To answer this question, first, we should be able to solve two main issues: (i) How to efficiently determine whether an error occurs when data integrity is calculated?; and (ii) how to prevent the diffusion of an error caused by a miscalculation of data integrity?

Traditionally, state-of-the-art techniques intended to answer these questions base their approach on redundancy by including only one of the following main elements: hardware, time, or information [10,11]. Hence, in hardware redundancy, the physical ciphering modules (processors, memories, multipliers, etc.) are replicated, generating several hashes at the same time. Time-redundancy architectures execute the same operation for providing multiple copies of the hashes at different times. Finally, information redundancy adds check data to verify hash correctness before using it and, in some cases, even allowing the correction of some erroneous hash [12,13,14]. Each architecture implementing some kind of redundancy presents advantages and disadvantages concerning power consumption, amount of hardware resources, and performance (throughput and efficiency). Hence, to provide a fault-detection architecture capable of being used in several environments, it is essential to balance system overload in terms of energy consumption, processing delays, and performance, among other parameters.

In this paper, we propose an innovative hybrid-redundancy hardware architecture for the cryptographic algorithm SHA-2. This architecture is designed to improve error and fault detection. In the following, we list our main contributions:

- To the best of our knowledge, pipelining has not been previously used to perform different hash calculations with a redundancy topic. Pipelining is traditionally used to process several instructions or operations at once, and its use in redundancy and cryptography is a relevant part of the innovation of this work. Therefore, we present a hybrid architecture, which presents a 3-stage pipeline structure, implementing hardware and time redundancy. We analyzed hardware resources and performance in the distribution of the processed elements within each stage and the balance of critical path. Besides, we increased CPU performance at a certain clock speed, defining a transformation in data flow in several sequential phases where the input of each stage is the output of the previous one;
- From a pipeline design perspective, data transmission is improved by eliminating related risks and conflicts, using registers in each stage for processing the hash functions, which perform multiple passes to the data to obtain their final hash. By adding an error verification module, based on a voting scheme, we were able to determine whether an error occurred or, otherwise, the output data were correct. Designing an architecture with a pipeline scheme and redundancy processes is not straightforward since several design problems, related to data dependencies, must be solved along the way;
- Another contribution is the creation, analysis and demonstration of both fault tolerant and non tolerant architectures, which lays the ground for future research and studies towards understanding the advantages and disadvantages they present, also allowing comparison with other works. The selection of the architecture, along with its design and implementation, were evaluated and analyzed using various FPGA technologies (Spartan-7, Artix-7, Kintex-7, and Virtex-7). The Virtex-7 implementation was the one that achieved the best results of all the alternatives, with a throughput of 441.72 Mbps and 2255 LUTs, presenting an efficiency of 195.8 Kbps/LUT. It is true that the examined technologies are produced by the same company; however, it must be emphasized that each of them is different from the rest, as mentioned in [15]. Hence, regardless of whether they are from the same family or not, their logic blocks can be quite different in their structure.

The content of this paper is divided as follows. Section 2 presents a background about the SHA-2 hash function, fault-detection schemes and pipeline. In Section 3, several related works are presented and explained. Next, Section 4 presents the proposed hardware architecture for fault-detection. We present the results and their comparison with other techniques in Section 5. The discussion of the results is presented in Section 6. Finally, the main conclusions drawn from the results of this paper are presented in Section 7.

## 2. Background

This section presents some definitions about the SHA-2 algorithm and error detection/ correction. The description of the fault-detection context for applications such as cyber-physical systems, vehicular communication networks, and Industry 4.0, to assure higher performance, is presented as well. Besides, we describe FPGA optimization techniques for hardware design.

### 2.1. SHA-2 Algorithm

One of the main types of cryptographic algorithms is the hash function, where SHA-2 is a one-way cryptographic function, which is one of the most used. It receives a message as input and returns a fixed-length digest, which is used to authenticate the original message, as output. Hashing is destructive, and data are lost. Therefore, it is irreversible, and the original message cannot be retrieved from its hash.

The SHA-256 hashing algorithm takes an original message and divides it into 512-bit (i.e., 64 bytes) input blocks, processes them, and generates a 256-bit (32 bytes) output value. Specifically, first, the original message is appended and padded, generating only *n* 512-bit blocks. Secondly, eight hash values and 64 round constants are initialized. Thirdly, each 512-bit block is processed accordingly to create a message schedule and compute operations such as XOR, rotations, additions, and others 64 times. Finally, the message is compressed because the 256-bit hash outputs of each block in the previous step are the new eight hash values for the next block; when all the 512-bit input blocks are processed, a final 256-bit hash value is provided (more details in [9]).

### 2.2. Fault-Detection

Fault tolerance can be understood as the capability of a system to perform regularly, even in the appearance of errors. For example, a crash in the resources or the presence of faults [16]. A fault-detection mechanism, in general, should have the following features:

- A reliable integrity calculation process, based on hash assessment, capable to determine whether the information is faulty (no matter the reason) or reliable (i.e., if the computed hash is valid). The reliability of the result of the fault-detection mechanism is basic for decision making;
- A light computation process that avoids excessive energy consumption. This is important, especially when the implementation is made on a device with limited resources, such as a wireless sensor or a mobile phone;
- A fast assessment of data validity, which makes the mechanism applicable in real-life situations, where a fast response to errors is mandatory. An in-time error-detection mechanism allows taking fast actions like re-fetching the corrupted data or flagging the possibility of a compromised communication channel.

## 3. Related Work

Several works have been proposed to prevent and detect transient errors or faults for applications at the hardware level. In this sense, implementations of hash-function have been used to provide integrity and reliability over the data transmitted and generated for communications. Nevertheless, given the way SHA algorithms work (which consists of a high-diffusion iterative structure), the presence of small errors in the hash calculation will result in several ones in the final hash [1]. In the following, some outstanding works that tackle this problem are presented.

Ahmad and Das proposed, in [1], an error detection approach using parity codes and hardware redundancy. Their performance metrics indicated that their limitations were hardware overhead and short delay overhead. They did not introduce a pipeline method to optimize the architecture. Besides, their scheme did not carry out the correction of the detected error.

Bahramali et al. proposed, in [17], a fault diagnosis method for SHA-1 and SHA-512, focused on detecting transient and permanent faults. The proposal used time redundancy and a pipelining method. Their work included, in the critical path of the SHA hash functions, the addition operation, then they used a subtraction as the inverse function. This process was used for redundancy, detecting both permanent and temporary faults. They observed that traditional time redundancy approaches do not have this capability because they only detect transient faults.

A Totally Self-Checking (TSC) design was introduced for the SHA-256 hash function by Michail et al., in [18], suitable for harsh environments, focused on detecting erroneous bits (odd and even numbers), if they are appropriately propagated. They observed that ASIC technologies are more adequate than reconfigurable devices, such as FPGAs, for TSC. Besides, in [19], they presented a comparison between two hash functions, SHA-1 and SHA-256. In this case, they analytically described the development process for each component of TSC as compared to the Duplicated-with-Checking (DWC) alternative. Their conclusion was that, in comparison with DWC architectures, TSC cores have more benefits in terms of area, throughput/area, and power consumption.

Kahri et al., proposed, in [20], an FPGA fault detection scheme to protect the SHA algorithm against attacks based on hybrid (hardware and time) redundancy. The trade-off between security and cost of implementation is an advantage of this approach.

Algredo-Badillo et al. [21] proposed a pipeline scheme for the AES symmetric cryptographic algorithm, unlike the architecture proposed in this paper, which is focused on the SHA-2 cryptographic hash function. Additionally, while the AES architecture uses a structure with 5-pipeline stages, our architecture uses 3-pipeline stages.

## 4. Proposed Hardware Architecture

As mentioned before, SHA-256 takes a message and generates a digest, where the message is padded and divided into 512-bit message blocks, each of them going through 64 processing rounds. In this section, two hardware architectures are proposed: (a) a simple hardware architecture, which has an iterative structure and does not detect errors; and (b) a hybrid-redundancy hardware architecture, which is fault-tolerant and based on the simple hardware architecture.

**Figure 1:**
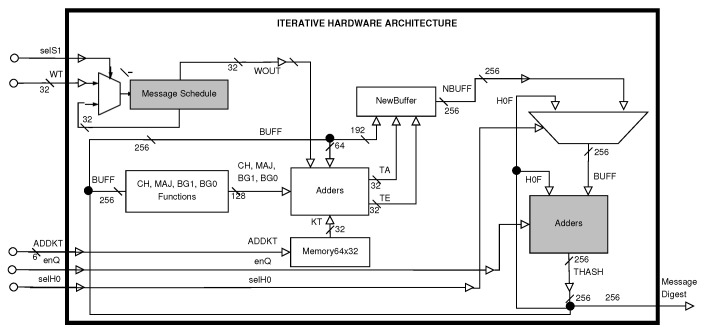
Iterative hardware architecture diagram without fault detection or pipeline structure.

The first proposed architecture is a simple one, which processes 512-bit message blocks during 64 clock cycles, and is not designed to detect faults, processing one 512-bit message block at a time, given its iterative structure; it also has a final message digest, which is generated after 64 clock cycles (see Figure 1), for a given message block. The gray modules in the simple architecture correspond to the data storage for the input message and the intermediate hash.

**Figure 2:**
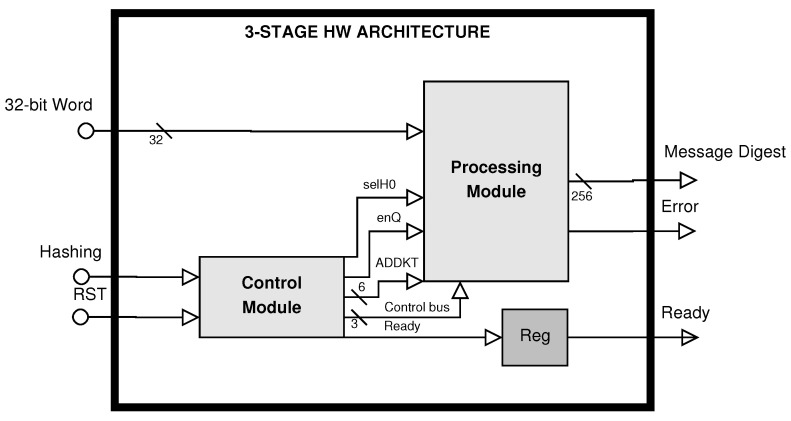
3-Stage hardware architecture block diagram with error correction.

The second proposed architecture is the hybrid-redundancy one, which is based on computing the SHA-2 algorithm three times on the same 512-bit message block. The block diagram of this architecture (see Figure 2) has two main modules: (1) the control module and (2) the processing module. The algorithm defines 64 rounds; consequently, the proposed architecture makes different operations and requires 196 clock cycles for processing the first 512-bit message block, and 195 clock cycles (latency) for the remaining 512-bit message blocks, and all of them constitute the complete message. This latency is present because the 3-stage pipeline structure requires 3 clock cycles for computing one round defined by the SHA-256 algorithm, where the key idea is to compute three times the message digest. Additional clock cycles are necessary for initialization, data storing and output generation.

On the one hand, the processing module takes 32-bit words from the given 512-bit message block and executes several computations at the same time throughout the pipeline structure. At the end of the 196 or 195 clock cycles (depending on the case), this module has three registers with the hashes of the three pipelines, which are compared for determining whether an error occurred or not. The proposed hardware architecture takes into account three cases: (a) if the three hashes are different, then an error is produced, the error signal is set, the message digest is incorrect, and it must be ignored; (b) if two hashes are equal, then a fault is detected, but the architecture corrects and generates the exact output, the error signal is reset (indicating there is no error) and the message digest is correct; and (c) if the three hashes are equal, then there is no error, the error signal is reset, and the message digest is correct. In summary, if an error is detected, this can be solved or not; if this is not solved, then the 256-bit output hash of this module must be ignored; otherwise, the output hash is correct. On the other hand, the Control Module enables the dataflow of the first module. In the next subsections, more details are provided for both modules.

### 4.1. *Processing Module*

**Figure 3:**
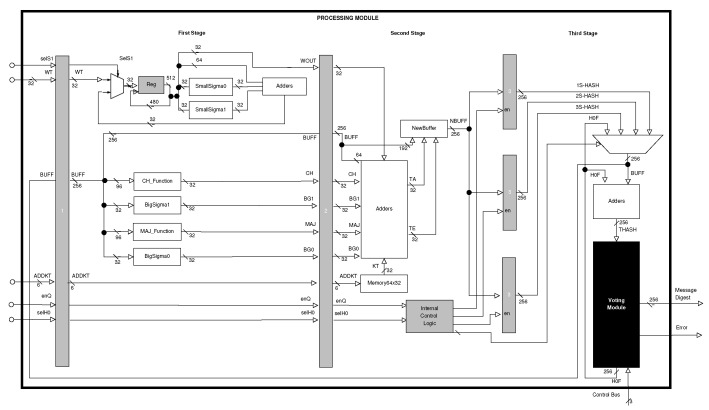
Block diagram of the processing module for the hybrid–redundancy hardware architecture.

The Processing Module has three stages (see Figure 3), which try to balance the critical path. This module represents the pipeline architecture and shows the three stages, which are separated by registers (gray blocks) that are populated using data in sequence, that is, the three replications of a same message block.

**Figure 4:**
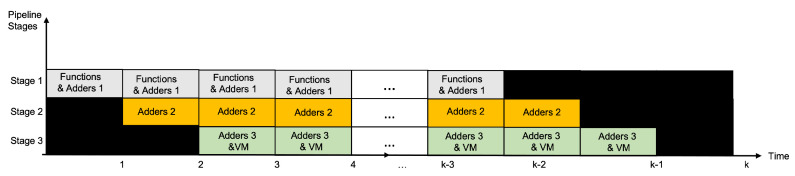
The three stages of the proposed pipeline structure.

The general process for a message block can be explained as follows (see Figure 4):

- In the first clock cycle, the block is fed to the processing module and the first stage, *Functions&Adders1*, is computed (six functions and the sums);
- In the second clock cycle, the output of the *Functions&Adders1* stage is input to the *Adders2* stage and the first stage is fed again with the same block (replicated block);
- In the third clock cycle, the output of the *Adders2* stage is input to the *Adders3&VM* stage, the output of the *Functions&Adders1* stage goes to the *Adders2* stage, and the first stage is fed finally with the replicated block;
- From the fourth cycle to the 194-th cycle, the three repetitions of the same message block will be moving from the first to the second stage, from the second to the third stage and from the third to the first stage. This is focused on calculating the 64 rounds of the algorithm for each replication of the message block;
- Finally, in the 195-th clock cycle, the three hashes are obtained and the outputs can be generated by comparing, evaluating and selecting the registers.

The previous process is executed for each message block for which its hash value must be obtained. The replications of a second block (or remaining blocks) of the message can be entered, maintaining the pipeline as busy and improving performance and efficiency with a latency of only 192 clock cycles, which represents the best case for metrics and results.

Next, each stage is described to explain the inputs, outputs and detailed behavior of the architecture, where each stage executes different tasks (see [9]):

#### 4.1.1. First Stage

It performs message scheduling, also executing the following four functions: Ch(x,y,z), Maj(x,y,z), $\Sigma_{0}^{256}$, and $\Sigma_{1}^{256}$.

In this stage, for message scheduling (at the top left of Figure 3), a register is used for storing 512 bits (sixteen 32-bit words) during sixteen clock cycles, for the first sixteen rounds. Later, for the remaining forty-eight rounds, it is necessary to compute new 32-bit words and functions (SmallSigma0$\sigma_{0}^{256}$ and SmallSigma1$\sigma_{1}^{256}$) and adders are implemented.

In this same stage, several temporary variables are computed (CH, Maj, SG0, and SG1), which are used in the next stage for computing the new state buffer (NBUFF). The input for these functions is the initial hash or intermediate hash from the previous round.

#### 4.1.2. Second Stage

It computes the new state variables (A,B, …, H), along with the internal control logic for registering three dataflows of the intermediate message digest. For this computing, the round constant, the round word, and the four outputs of the first-stage functions are used.

The second stage retakes a great amount of the variables generated in the previous stage for computing the new state buffer. These variables are: (1) one round 32-bit word (WT); (2) four temporary variables (CH, Maj, SG0, and SG1); (3) one 256-bit state buffer (BUFF); and (4) one address (ADDKT) for obtaining the round constant (KT). Additionally, the second stage must store the new state buffer in one of the three registers because this carries out the computing of the three different processes (by the pipeline structure) of the SHA-256 algorithm.

**Figure 5:**
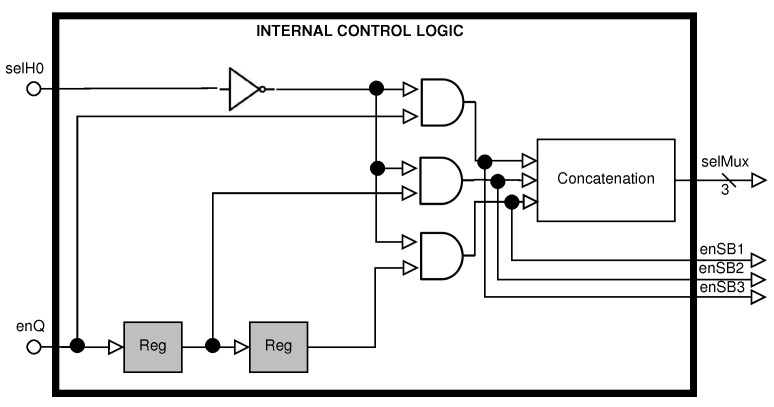
Block diagram of the Internal Control Logic.

The selection of the register for storing the state buffer is executed by the Internal Control Logic (see Figure 5). This has two signals provided by the Control Module, whose logic allows the selection of the register according to the data flow of the three pipeline processes.

#### 4.1.3. Third Stage

It calculates the final, intermediate and replicated message digests. In this architecture, padding is not considered in pre-processing; for that reason, the input message must have a length less than $2^{64}$ and be a multiple of 512 bits. This message is divided into 512-bit blocks, and the architecture can process one or more 512-bit blocks (*N* 512-bit blocks) for obtaining the final digest of the same message. Each 512-bit block is sequentially processed during several clock cycles and divided into sixteen 32-bit words. Additionally, a block is processed three times by the proposed hybrid architecture, where the Voting Module selects an output from the three final hashes; finally, it decides if there is an error or not.

The third stage considers several situations: (1) selection of the initial state buffer (constant values when the first 512-block is processed, or message digest from the previous 512-bit block when the message is constituted by several 512-bit blocks); (2) update of the intermediate state buffer (this occurs when it is processed in the internal rounds, from the 2nd to the 63rd, for the three computing lines); (3) computation of the intermediate message digest (from all the 512-bit blocks, except for the last one), and computation of the final message digest (from the last 512-bit block of the message).

**Figure 6:**
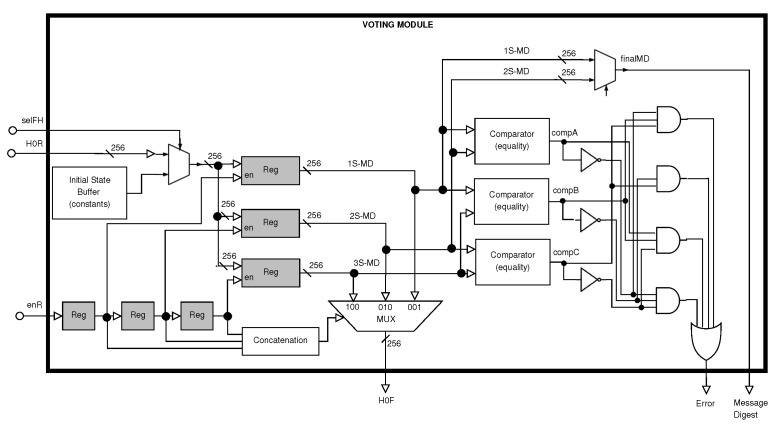
Block diagram of the Voting Module.

In the third stage, the Voting Module stores the three final digests of the message, computing the 64 rounds defined by the SHA-256 algorithm (see Figure 6). Moreover, this module compares these three values for generating the final message digest. Finally, it also generates the Error signal, which is a flag that indicates if the final message digest is validated or not.

The *Voting Module* generates: (1) the H0F bus for intermediate message digest or initial message digest; (2) the final Message Digest bus for the digest of the complete message; and (3) the Error signal for providing the flag informing whether the final message digest is correct or not. This flag is generated accordingly to compare pairs of message digests from each stage (see Table 1). This shows that the compA signal compares 1S-MD and 2S-MD (message digests from the first stage and the second stage, respectively), and it is high if they are equal and low otherwise. compB compares 1S-MD against 3S-MD and compC compares 2S-MD against 3S-MD.

The truth table for generating Error and *selMUx* signals and *selMUx* are shown in Table 2, where the first signal is low when two or three stage message digests are equal, and the second one selects 1S-MD or 2S-MD, which is the output bus for the Message Digest from the proposed hybrid architecture. 3S-MD is ignored because the possible outputs of the message digest indicate that selecting 1S-MD or 2S-MD is sufficient for generating the correct output bus. This analysis shows that logic is simplified due to the selection of only two buses (multiplexer and control logic are simpler).

The architecture proposed in this work provides some level of inner fault-tolerance. It means that if two hashes are equal and the other one is different, then a fault has occurred; in this case, the architecture is able to catch this fault and correct it; hence, the Error output signal is reset and Message Digest has one validated hash. This situation happens three times: cases “001”, “010”, and “100”, when one comparator is high. If the three hashes are different, case “000” happens, then a fault is discovered, the architecture cannot recover, Error output signal is set, and Message Digest must be ignored. Moreover, there are three states that cannot be reached: cases “011”, “101”, and “110”, because they indicate contradictions; for example, case “011” means that 1S-MD and 2S-MD are not equal, but the pair 1S-MD and 3S-MD is equal, in addition to the pair 2S-MD and 3S-MD, which cannot be true. In these situations, the Error signal is set, and the output Message Digest does not care (*X* is Don’t care condition). Finally, in case the three hashes are equal, case “111”, the fault is not discovered, then the Error signal is reset, and the Message Digest is correctly computed.

### 4.2. Control Module

**Figure 7:**
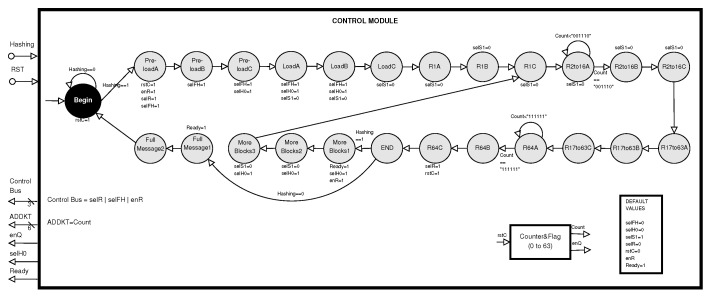
State diagram of the control module.

The *Control module* is in charge of the data flow, which enables dataflow in the Processing Module (see Figure 7). This control module is a DFA, based on a Moore machine, where the outputs of each state are listed below it.

The logic of the ControlModule is the preloading of the data (configuration values) and the loading of the input message (512-bit blocks), tasks executed by the states from Pre−loadA to LoadC. After the first round is executed (states R1A, …, R1C), rounds from 2 to 16 (states R2to16A, …, R2to16C), rounds from 17 to 63 (states R17to63A, …, R17to63C), and the final round (states R64A, …, R64C). Next, two situations can happen: a) there are more 512-bit blocks from the same message, hence the process starts by using an intermediate hash (states MoreBlocks1, …, MoreBlocks3), or b) the complete message has been processed and there are no more 512-bit blocks (states FullMessage1 and FullMessage2), thus the automaton is initialized and the new state is Begin.

The ControlModule manages: (a) the memory that stores the round constants through ADDKT addresses; (b) the multiplexer, which selects the initial hash or the intermediate hash through selH0 and enQ signals; (c) the multiplexer and registers for selecting and saving the three computed hashes through selFH, H0R, and enR signals; (d) the counter for the 64 rounds defined by the algorithm through the rstC signal; and (e) the multiplexer for obtaining external or internal 16-bit words through the selS1 signal.

## 5. Results and Comparisons

This section describes the following results: (a) the evaluation of the hybrid architecture with fault detection in the presence of test vectors and (b) the evaluation of its implementation on FPGAs, along with their comparison with architectures presented in related works.

**Figure 8:**
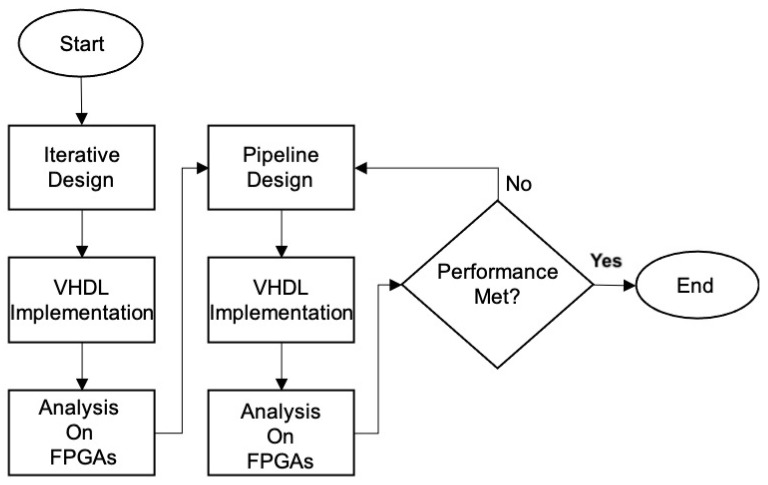
Methodology for developing and evaluating the hybrid hardware architecture.

The methodology used in this research is based on obtaining an optimized architecture focused on the minimum amount of specialized modules, providing an iterative design, where there is a control machine that manages data flow in all modules operating concurrently and in parallel this machine is also in charge of handling the algorithm process. The methodology is shown in Figure 8.

This design is implemented in VHDL and analyzed in FPGA devices using Xilinx tools. The results of this iterative design are also used for comparison purposes against the hybrid architecture with error correction. Once the iterative architecture presents optimal values (which occurs when there is a trade-off between a minimum number of clock cycles/hardware resources and high throughput) the critical paths and long paths are analyzed to decide where to place the registers of the pipelined stages. This process is not obvious or straightforward since the algorithm has an iterative process at the data level (data are operated in different branches). At the block level, each block is processed during 64 rounds as defined by the standard. At the message level, the full message is split into blocks, where each block result is chained to generate a fixed-length hash, regardless of the size of the original message. Additionally, the architecture is designed and modified to implement two redundancy schemes: time and hardware (hence the term hybrid architecture) using an error-correction scheme. Then, several designs are required to find the optimum, focused on the same type of trade-off analysis of the iterative design.

Different architectures were designed to achieve the optimal trade-off, which is decided until each of them was implemented in VHDL and evaluated on FPGA devices, as shown in Figure 8.

### 5.1. Analysis on Pipeline Architecture

This section describes the main advantages of the hybrid pipelined structure, measuring and comparing it against iterative and parallel structures. The proposal consists of combining attractive features of hardware and time redundancies. Specifically, we use the same hardware resource at different times (as defined in time redundancy) and simultaneously process several blocks (as defined in hardware redundancy). We integrate both redundancies using a pipeline structure.

In the following, we present a formal analysis of the hybrid pipelined structure, focusing on three cases: (a) hardware redundancy; (b) time redundancy; and (c) computation of the proposal in the hardware architecture (hybrid redundancy).

Let (*B*) be a SHA-256 simple iterative architecture (see Figure 1) requiring a power $P_{1}\left(t\right)$ and an energy $W_{1}\left(t\right)$, which can be represented as follows:

(1) $$\begin{array}{c} P_{1}\left(t\right)=p\\ W_{1}\left(t\right)=\int P_{1}\left(t\right)dt=\int pdt=pt+c_{1}. \end{array}$$

**Figure 9:**
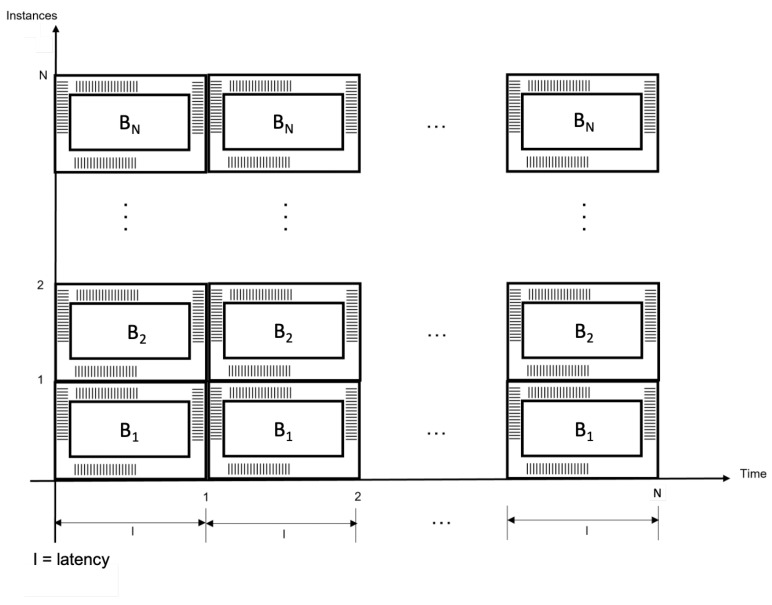
Hardware redundancy enables *N*$B_{i}$ blocks, for 1≤i≤N, where $N\in Z^{+}$, to operate in a parallel way during a given latency *l*.

At this point, power consumption is constant for an instant and over time, while energy consumption is variable ($pt+c_{1}$) according to time. *B* requires a latency *l* to obtain a correct hash value, which corresponds to a message block. The replicas of this architecture *B* are labeled as $B_{1},B_{2},B_{3},\ldots$, etc. Additionally, the SHA-256 hybrid redundancy hardware architecture (see Figure 3) is labeled as $B^{\left(h\right)}$ and its replicas are labeled as $B_{1}^{\left(h\right)}$, $B_{2}^{\left(h\right)}$, $B_{3}^{\left(h\right)}$, …, etc. The latency required by this architecture is represented by $l^{\left(h\right)}$. In this sense, three different types of analysis are carried out in this work:

Case (A) Architecture for hardware redundancy.In this case, hardware redundancy is implemented by means of the $B_{i}$ blocks (modules $B_{1},\;\ldots ,\;B_{N}$) for 1≤i≤N, where $N\in Z^{+}$. Using this scheme, parallel and concurrent processing is successfully achieved, which is shown in Figure 9. This figure reports high throughput, although all the devices are operating at the same time and each device requires a specified latency to process some data block.

This proposal, as can be seen, sacrifices energy and power consumption because all *N*$B_{i}$ modules are present; hence power consumption and energy are high and depend on *N*, as can be verified in Equation (2).

(2) $$\begin{array}{c} P_{hw}\left(t\right)=Np\\ W_{hw}\left(t\right)=\int P_{hw}\left(t\right)dt=\int Npdt=Npt+c_{hw}. \end{array}$$

**Figure 10:**
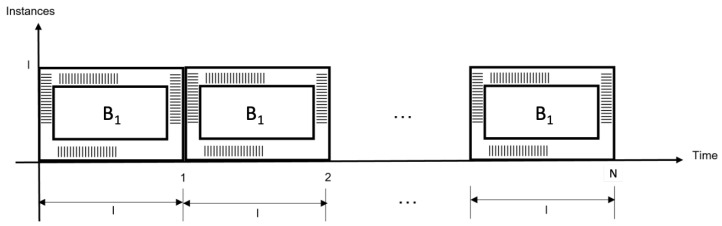
Time redundancy requires one module ($B_{1}$) to be sequentially executed in *N* clock cycles.

Case (B) Architecture for time redundancy. As opposed to the previous case, this proposal processes a single data block, using a single module *B*, during *N* time (included and represented by the time redundancy, named *N*). This is required in module ($B_{1}$) from iteration 1 to iteration *N* (the latency needed to process each data block is represented by each time segment), which can be verified in Figure 10. This proposal requires fewer hardware resources; however, throughput decreases as a consequence.

The advantage is that a module or instance does not consume a high power *p*, although the required energy is high as time goes by, since it requires more usage time, depending on latency and the number of redundant computations (this is modeled in Equation (3)). This situation represents a drawback, because throughput is decreased.

(3) $$\begin{array}{c} P_{ti}\left(t\right)=p\\ W_{ti}\left(t\right)=\int P_{ti}\left(t\right)dt=\int pdt=pt+c_{ti}. \end{array}$$

**Figure 11:**
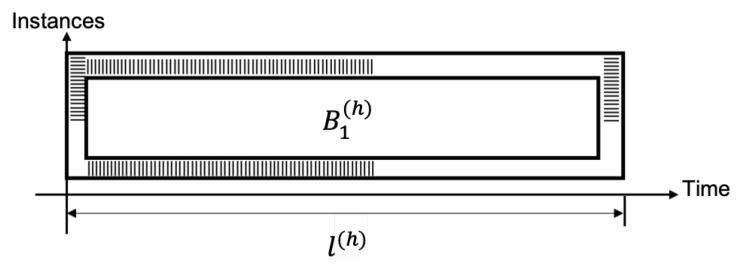
Hybrid redundancy in our proposal needs one block $B_{1}^{\left(h\right)}$, requiring $l^{\left(h\right)}$ clock cycles (latency).

Case (C) Architecture for hybrid redundancy.The proposed hybrid architecture defines an instance $B_{1}^{\left(h\right)}$ that operates several times with the same data block (for example, *N* times), reporting a greater latency $l^{\left(h\right)}$ when compared against *l*. Unlike case A, where *N* blocks are used, this architecture requires only one instance ($B_{1}^{\left(h\right)}$) to be used during a given time; and as opposed to case B, where a block is used several times, block $B_{1}^{\left(h\right)}$ is occupied just once in this architecture (with its corresponding latency). The corresponding latency is proportional to *N*, which can be verified in Figure 11.

In this case, there is a power consumption *p* required by the device. Energy consumption can be inferred from the time a data block takes to be processed.

(4) $$\begin{array}{c} P_{hy}\left(t\right)=p\\ W_{hy}\left(t\right)=\int P_{hy}\left(t\right)dt=\int pdt=pt+c_{hy}. \end{array}$$

Many factors affect energy and power consumption, such as resources for placing, routing, temperature, designs, devices, etc. In a constant scheme, if those factors do not affect, consumption is very similar to case B. Nonetheless, this alternative presents a number of advantages, which are that because the internal processes are shorter, the data travels shorter paths (it is easy to verify this behavior by observing the critical path and the minimum clock period). To summarize, this architecture, $B^{h}$, has an energy consumption similar to case B but with shorter critical paths. On the other hand, case A generally shows minimum energy consumption and poor performance; however, passive and active hardware resources require the same energy, regardless of whether the instances or blocks have a standby process. It must be noted that, if they are put in a standby state, then there is no need to have several instances of the block; hence, there would be a noticeable performance gain for most of the applications. It is important to highlight that an unrolled architecture can improve the results of this proposal, although this must be further analyzed.

### 5.2. Evaluation Metrics on Implementation

This section is devoted to the presentation of the implementation results for the proposed hardware architecture for its validation and prototyping. There are different FPGA manufacturers, and each one features several technologies, architectures, configurations, families, and devices for the development of software and hardware implementations. Each FPGA family is designed for a particular target market, offering at least three options for developers: low, medium and high range. Testing with a vast spectrum of manufacturers and technologies was not the central objective of this work and is impractical. However, we compared one type of manufacturer considering four different FPGA families and the three options offered to developers [22]. In this sense, the objective of the implementations is to show an analysis of the scope of the proposed hybrid–redundancy architecture, where we can observe the requirements of this fault-tolerant architecture, concerning design and implementation results, in addition to an analysis for comparing fault-tolerant architecture and non-tolerant architecture. We also understand that the architecture of each manufacturer is very different and they must be evaluated, but our results show that there is no dependency on specialized resources and common hardware resources are required for the fault-tolerant encryption process. In this way, the proposed architecture is synthesized, mapped, placed, and routed for different FPGA technologies: Spartan-7, Artix-7, Kintex-7, and Virtex-7 devices with the Vivado 2019.2 design tool. The implementation considers real-time operation conditions (the design conformance test data are used for this purpose) for simulation and verification.

These architectures are evaluated using several metrics, some of them provided by the Vivado tool (number of slices, LUTs, Flip-flops (FFs), BRAMs, DSPs, and IOBs, as well as the minimum clock period), some other metrics (throughput and efficiency) are obtained by performing some calculations, as explained next. All these metrics are used to analyze different trade-offs. The processed bits per second (bps) are used to compute throughput (we can obtain the clock frequency when the architecture is implemented on different FPGA devices). This can be observed in Equation (5).

Implementation efficiency is the other metric. For a given implementation, it is defined as the ratio between the achieved throughput and the area or amount of hardware resources used for the implementation. The number of LUTs an implementation consumes (bps/LUT) as well as the number of FFs it uses (bps/FF) are two examples of efficiency. This is reflected in Equation (6).

(5) $$Throughput=\frac{Data\_block\_size}{\left(Clock\_time)(Clock\_cycles\right)};$$

(6) $$Efficiency=\frac{Throughput}{Number\_of\_Slices}.$$

Next, two types of analysis of results are presented: (1) the comparison between the simple SHA-256 architecture and the hybrid SHA-256 architecture with fault detection and (2) the comparison with related works.

The iterative structure in the simple SHA-256 architecture (see Figure 1) processes a 512-bit message block during 64 clock cycles. This architecture does not detect faults and is a basic element for the design and development of the fault detection SHA-256 hybrid-redundancy structure, which has a 3-stage pipeline architecture.

For the comparison between the two SHA-256 architectures, i.e., the simple and the hybrid one with fault detection, implementation results appear in Table 3 for the former, and Table 4 for the latter. These tables show the hardware resources required for the corresponding implementation (LUT, LUTRAM, FF, IOB, and BUFG), along with the physical resources needed in terms of clock period (the tool itself provides them all); used data size and latency, both of them characteristics of the design, are also shown. Finally, the performance measures, throughput and efficiency, shown in Equations (5) and (6), respectively, are presented as well. The critical path time corresponds to the clock period, i.e, the minimum required clock period used to determine the maximum clock frequency, which is, by definition, the inverse of the minimum clock period; this establishes the throughput and efficiency values of the diverse implementations.

There are two points worth mentioning, according to design and implementation. On the one hand, the proposed designs reflected three constant parameters: (1) latency is 195 clock cycles for tolerant architecture and 64 clock cycles for non-tolerant architecture; (2) data size is 512 bits for both architectures; and (3) tolerant architecture has 293 input/output ports (I/Os) and non-tolerant architecture has 292 I/Os. On the other hand, the implementations of those architectures show two constant parameters: (1) the number of FF is 2516 for tolerant architecture and 753 for non-tolerant architecture; (2) 64 LUTRAMs are used in both architectures; and (3) tolerant architecture requires 2255 LUTs and non-tolerant architecture needs between 1338 and 1350. The results of the implementation depend on the manufacturer’s FPGA architecture and the tool (and algorithms) for synthesis, place and route.

The hybrid architecture occupies approximately 68% more LUTs than the iterative architecture. It also uses 2.34 times more FFs (they are required for the registers of each pipeline stage and other extra modules needed for error correction). Both architectures use the same amount of LUTRAM because they store 64 round constants, and the hybrid architecture requires one additional IOB (for the Error signal) and one more BUFG.

The design of the hybrid architecture tries to balance the paths of each stage of the pipeline structure, and several modules are added to the simple architecture, where the critical path time is shorter except for the implementation in Spartan-7. Even though both architectures process 512-bit message blocks, they differ in latency since the simple architecture requires 65 clock cycles, while the hybrid one uses 195.

Table 4 presents unreal throughput, which is computed based on the 1536-bit size of the input data (three 512-bit message blocks), although the 512-bit bus is the real size. This throughput would apply if the hybrid architecture were used in a 3-stage pipeline structure with three communication lines (three 512-bit message blocks, i.e., an input data size of 1536 bits), but in the proposed situation, the hybrid proposal is utilized for detecting and correcting faults, processing a 512-bit message block using a 3-stage pipeline architecture. The iterative architecture performance is very similar to the unreal throughput and efficiency of the hybrid proposal, which is achieved for the design of the simple architecture (see Figure 1), requiring fewer modules and presenting a balanced critical path. Considering a real situation, the throughput of the simple architecture is almost three times higher than that of the hybrid architecture, and this also occurs for both efficiencies (Mbps/LUT and Mbps/FF).

The results of the comparison with related works are shown in Table 5. We are conscious that, since different FPGA technologies are used, this comparison cannot be considered to be fair. These results, however, provide us useful insights on the design techniques as well as the implementation results. All the works reported architectures on Virtex technology, excepting [1], which uses Altera technology. The presented implementations are SHA-1, SHA-256, and SHA-512 functions. Most works present two architectures (with and without fault detection). Disregarding [20], the architectures with fault detection implement hybrid redundancy (each work uses two types of redundancy), where hardware redundancy is a common technique. In our case, these architectures are named simple (without fault detection) and hybrid (with fault detection).

The architectures reported in [1] presented two SHA-512 hardware implementations—one of them was simple and the other was based on parity coding and hardware redundancy—the latter focused on single, permanent and transient faults. In comparison with our proposal, they used more than twice the hardware resources, which can occur given that the authors implemented the SHA-512 algorithm, although they used parity coding. In [17], a hardware architecture based on time and hardware redundancy was presented and considered permanent and transient faults. The authors implemented rounds for the SHA-1 and SHA-512 algorithms, where architectures with error detection reported a decreased throughput, and the SHA-512 architectures were faster than the SHA-1 architectures. Although using different technologies, the required amount of hardware resources was very similar to that of our proposal, even when they only implemented round computations.

In Kahri et al. [20], the authors presented a hardware architecture based on time-redundancy, which was designed against fault injection attacks. Using Virtex-II pro, the authors implemented the SHA-512 algorithm, and compared architectures with and without fault detection. The amount of resources used and the clock frequency were very similar to our proposal. Finally, in Michail et al. [18], the authors reported a hardware architecture using information redundancy and hardware redundancy, implementing the SHA-256 algorithm as in our proposal, even though they used Virtex-5 technology. They required more than 4 times the hardware resources and their implementation operated at lower frequency, although they reported the highest throughput. While similar throughput would be expected, this was not the case, and details about latency were not provided, hence no comparisons could be made on this point.

### 5.3. Analysis for Fault Detection

The proposed architecture was evaluated using a text file containing 5000 test vectors. The test bench in VHDL was used to apply these vectors at simulation time (this was done using the Vivado tool). The test vectors are made up of: (1) the multiple message organized in 512-bit blocks; (2) the control buses required by the control module for managing the data flow; (3) the right bit hash result; and (4) a 32-bit output, required for modifying certain internal computations and emulating external faults or internal faults.

**Figure 12:**
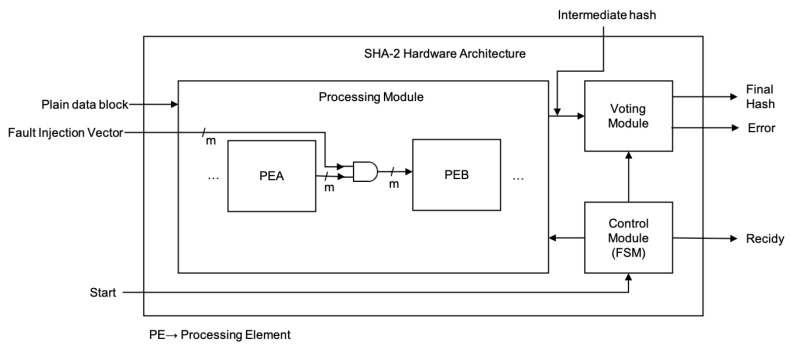
Modifications made to the hardware architecture in FPGA, only to evaluate, in a synthetic context, by injecting errors.

The 32-bit sub-vector, mentioned in 4, is key for this evaluation, since it is required for carrying out the modification of many computations at different hardware modules. For testing purposes, the hybrid architecture was modified three times. These modified versions of the hybrid architecture were not implemented in an FPGA, because they were only used for evaluating fault detection. The three versions were altered by executing an XOR operation between the 32-bit sub-vector of point 4 and the 32-bit output of: (a) the BigSigma0 module of the first stage; (b) the TE bus of the second stage; and (c) the lower bits of the multiplexer of the third stage. Modified blocks were labeled PEB, whose input was altered by an XOR operation between the original input and the injected error, which could change the value of the data bus, according to the truth table (see modules in Figure 12).

Using the proposed hardware architecture without modifications and the three modified architectures, the vectors were used to execute four different runs, i.e., 20,000 tests were evaluated. The hybrid architecture without modifications (this one was implemented in an FPGA) was tested in the first epoch; for this purpose, one complete epoch of the dataset was applied and analyzed by the test bench (in this case, the 32-bit sub-vector was not utilized). The three remaining epochs were applied to the three variations of the hybrid architecture; in these cases, 15,000 communication faults were simulated using the 32-bit sub-vectors. These results showed that all faults (i.e., 100% of them) were detected using the hybrid architecture.

The four runs were essential for analyzing error injection since cryptographic algorithms have high diffusion. It means that a slight modification (from 0 to 1 or vice versa) in a single bit can generate a different value in the output bus (most bits change their value). Therefore, two cases of error injection were analyzed: stationary and permanent cases.

For the stationary case, the results of this analysis showed us that in case one or two registers were altered, it was possible for the architecture to recover 100% (the majority prevailed). However, in case four registers were altered, the architecture was not able to recover (this happened because there were different contents in most of the registers). Hence, the output signals “ready but with an error” were sent, which meant precisely the system did not recover; in such a case, the result must be recalculated or simply cannot be trusted.

For the permanent case, the same logical operators were used for generating permanent errors, which were maintained. Here, the test vectors were changed as in real contexts. In this case, there were situations where output signals were used to emphasize it was not possible for the architecture to recover from the error, and that, although the output was ready, it was not correct, which also meant it had to be recalculated. In three runs, 500 test vectors were used, and the architecture recovered whenever three or more registers, at the end of the pipeline, matched (majority). Persistent failures occurred when there was a signal introducing errors (this changed when the architecture was able to generate the required majority since this was the only way it could recover from the error).

The results of these evaluations showed that both stationary and permanent errors were determined in 100% of the cases, thanks to the hybrid architecture, although the architecture may or may not recover, depending on the similarity of the registers. Because the modifications derived from the injection depended on the high cryptographic diffusion as well as on which bit was corrupted (since this, at the same time, depended on whether it is affected by the logical operation or not), it did not always happen that it resulted in a modification of the output.

In the temporary injection experiments, the test vectors with error were applied in one or two clock cycles, whereas in the permanent injections they were applied using three or more clock cycles. As a consequence, in the former case, these vectors altered not more than two stage registers in the pipeline structure; this change depended on the high diffusion, whether the local operation modified the bits, and whether the injection affected the stored hashes at the end of the pipeline (see Table 6).

Fault detection reached 100%, but recovery was about 34.2%. The correction ratio increased, provided that fault was injected in just three clock cycles in different modules of each pipeline stage. Whenever the injection increased, the correction ratio decreased in contrast. The novel approach proposed in this work allowed us to obtain these results, also providing a more reliable strategy for detecting and correcting faults.

## 6. Discussion

We emphasize that the proposed architecture maintains the complete calculation of the SHA-2 algorithm, without modification, but the hardware resources of the architecture are used iteratively with feedback, maintaining the standardized security levels established by different state-of-the-art analysis. The architecture proposed in this manuscript is not supposed to be lightweight since it consists of a completely unrolled round, which is used 64 times in the case of the non-tolerant architecture (64-cycle latency) and 192 times in the case of the tolerant architecture (included in the 195-cycle latency). The first architecture is iterative and is used to obtain the hash in a common way, while the latter computes three hashes, by using the same modules and checking whether they are correct or an error occurred; in the latter case, it checks if it is possible to recover from the error (by means of majority vote), otherwise, the Error output signal is set.

**Figure 13:**
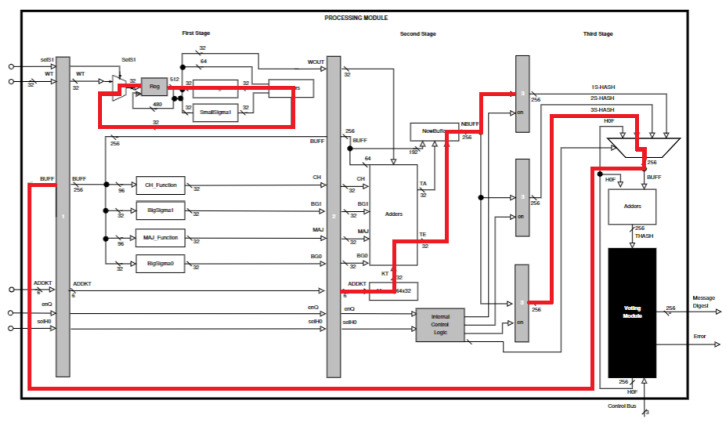
Critical paths in the architecture for each pipeline stage. Red lines indicate a path where hardware resources appear for both place and route processes.

Figure 13 shows the critical paths in each stage, which implies it is not possible to have large clock frequency values, because of the existing large paths. Red lines indicate a path where hardware resources appear for both place (hardware for algorithm logical and arithmetic calculations) and route (routing hardware) processes. Large paths can be reduced if more stages are used, which would need more pipeline registers and more proportional registers to store final hashes, depending on the number of stages; also, the voting module would increase complexity. For example, [21] describes a tolerant architecture for the symmetric AES-128 algorithm, where it is shown how complex the voting module becomes when a 5-stage pipeline is used, also showing there are options to reduce this combinatorial complexity. Increasing the number of pipeline stages is not the intention of this work, and this can be part of the future work, where exploring the relationship between stages and performance results can be an important part of the analysis; the intention of this paper is to explore, lay the ground, and innovate the pipeline technique in a redundancy process in the SHA-256 algorithm, which, to the best of our knowledge, has not been used in hash functions. Most pipeline systems achieve higher frequencies because all the dataflow allows dividing into a large number of stages, improving high throughput and frequencies; however, when prediction problems, jump/no-jump situations, halting, control risks, data risks etc. appear, a lot of the power of pipelining is lost. In cryptographic cases, such as that of SHA-256, there is a higher data dependency [23], which leads to zero advantages from using pipelining; in this situation, and in our case, it is not possible to apply pipelining to increase throughput, because high data dependency returns the system to the worst case scenario of pipelining because of data halt (i.e., the ciphering of a block must be finished before starting ciphering the next one; in other words, 64 rounds must be completely applied to cipher a block before starting the 64 rounds needed to cipher the next one).

The maximum frequency for some Virtex-7 devices is 700 MHz; however, a design will hardly reach that frequency since we should consider that the designs for some applications must use different configurations and number of gates with intermediate registers. The truth is that FPGA designs have different complexity, the algorithms that can be implemented are very diverse, and the architecture implementations reduce the maximum frequency range of the device [24]. Cryptographic solutions commonly represent a bottleneck; therefore, the need for research such as the one presented here, where the analysis shows the scope of our proposed architecture, which can operate according to its maximum frequency, performance, hardware requirements, and efficiency.

Traditionally, a pipeline structure has many stages, which means that high frequencies and high processing capabilities are achieved. Pipelining can hardly be used in cryptographic implementations since the operating modes of the cryptographic algorithms require finishing the encryption of a message block to start encrypting another block of the same message [25]. Pipelining is not practical for cryptographic solutions on a communication line. Instead, we use a pipeline for a single communication line, efficiently considering hardware resources, and with the ideology of redundancy. For example, suppose audio is sent on a communication line. In that case, this audio is separated into entire blocks and starts to be encrypted with SHA or AES in CBC/OFB/CFB/CTR mode. We remark that we must finish encrypting one block to start with another, so cryptographic architectures with many pipeline stages are useless here. The worst case occurs, as if all the pipeline stages were a single stage. Using *E* stages for the pipeline architecture would work if *E* independent communication lines were used. AES, in ECB and CTR modes, allows ciphering several blocks at once in a parallel fashion; although AES, in CBC, OFB, and CFB modes does not allow parallel ciphering, because it needs to finish ciphering one block before starting ciphering the next one. This completely affects a pipeline architecture, since the halting problem appears, which stops all the pipeline stages until the ciphering of a block is finished, i.e., there is no more advantage due to pipelining in working with several instructions or operations at once, more hardware resources are used and this is a disadvantage. This happens with any cryptographic hash function, such as SHA-256. We propose using redundancy and pipeline structures in any mode of operation for some symmetric cryptographic algorithm and any cryptographic hash function, showing the involved operations.

## 7. Conclusions

In this work, we present a novel hybrid architecture for SHA-256 implemented on a 3-stage pipeline structure that provides fault tolerance with iterative and feedback processes. Pipelining is traditionally used to process several lines of communication or several instructions at the same time, but for cryptographic algorithms it cannot be applied in a general way since there are three problems: (1) there is a high dependency on data; (2) there are iterative processes requiring several rounds; and (3) there is a need to encrypt one block to encrypt the next one (hash functions, CBC mode, blockchain, etc.), resulting in the pipeline not giving a clear advantage. In our proposal, we used pipelining to encrypt a block, solving the three previous problems within the same communication line and waiting to finish encrypting a block to start the next one, focusing on redundancy tasks. Besides, we analyzed hardware resources and performance to balance the critical path. It is important to highlight that fault tolerance increases the requirement of hardware resources for adding pipeline registers, and latency is higher because several stages, at different iterations, must be executed, reducing throughput and efficiency due to such an increment. Our architecture reported a throughput of 441.72 Mbps and 2255 LUTs, and presented an efficiency of 195.8 Kbps/LUT.

## 8. Future Work

As a future work, we suggest exploring a lightweight architecture, where the round is compacted into a Von Neumann architecture. One example of this is the architecture shown in [26], where latency is considerably increased and performance is reduced, but the use of hardware resources is improved, also reducing energy consumption. In that suggested future work, redundancy should be explored along with pipelining and lightweight architectures, to carry out research concerning the different design and implementation parameters, as well as to explain the behavior due to the combination of these techniques. Besides, we will focus on reducing power-draining by considering the idea developed by Pal and Mukherjee in [27]. They proposed a logic that when there was no fault in the system, instead of activating all of the modules, they only activated two of them. When any mismatch was found between these two modules, they activated the spare modules; their aim was to save overall power consumption and maintain system reliability. We will also evaluate the idea proposed by Wang and Liu [28] to improve the voting module. They recognized the cost of hardware redundancy in their work, so they proposed a scheme where less hardware was required. They used an algorithm to calculate the weight of the sensor signal based on the difference between the sensor signal and the analytical R-value, so that the voting value is closer to the actual value.

## Figures and Tables

**Table 1:** Resulting signals of the comparators for each pair of stage message digests.

Message Digest of Each Stage	compA	compB	compC
1S-MD	x	x	
2S-MD	x		x
3S-MD		x	x

**Table 2:** Combinations of inputs for *Error* and Message Digest signals.

compA	compB	compC	Error	Posible Output of the Message Digest	selMux	Message Digest
0	0	0	1	X	X	Don’t care
0	0	1	0	2S-MD or 3S-MD	1	2S-MD
0	1	0	0	1S-MD or 3S-MD	0	1S-MD
0	1	1	1	X	X	Don’t care
1	0	0	0	1S-MD or 2S-MD	0	1S-MD
1	0	1	1	X	X	Don’t care
1	1	0	1	X	X	Don’t care
1	1	1	0	1S-MD, 2S-MD or 3S-MD	0	1S-MD

**Table 3:** Implementation results for the simple SHA-256 hardware architecture with iterative structure.

Simple SHA-256 Hardware Architecture with Iterative Structure				
Range	Low	Low	Mid	High
FPGA technology	Spartan-7	Artix-7	Kintex-7	Virtex-7
Device	xc7s75fgga676-1	xc7a75tfgg676-1	xc7k70tfbg676-1	xc7vh870tflg1932-1
Clock period (ns)	9.386	10.305	7.153	7.039
Frequency (MHz)	106.54	97.04	139.80	142.96
Throughput (Mbps)	839.22	764.37	1101.20	1119.04
Efficiency (Mbps/LUT)	0.6272	0.5712	0.8163	0.8289
Efficiency (Mbps/FF)	1.1145	1.0151	1.4624	1.4861

**Table 4:** Requirements on FPGA for the SHA-256 hybrid-redundancy hardware architecture for detecting faults using a 3-stage pipeline structure.

3-Stage SHA-256 Hardware Architecture with Fault Detection				
Range	Low	Low	Mid	High
FPGA technology	Spartan-7	Artix-7	Kintex-7	Virtex-7
Device	xc7s75fgga676-1	xc7a75tfgg676-1	xc7k70tfbg676-1	xc7vh870tflg1932-1
Clock period (ns)	9.602	9.458	6.187	5.944
Frequency (MHz)	104.14	105.73	161.62	168.23
Unreal throughput (Mbps)	820.34	832.83	1273.14	1325.18
Throughput (Mbps)	273.44	277.61	424.38	441.72
Efficiency (Mbps/LUT)	0.1212	0.1231	0.1821	0.1958
Efficiency (Mbps/FF)	0.1086	0.1103	0.1686	0.1755

**Table 5:** Comparisons of hardware architectures between this and related works.

Work	Characteristics	Throughput (Mbps)	Objective
	SHA-512: 5038 LEs, altera EPIS20F780C5, 80 clock cycles	372	Error detection
[1]	SHA-512: 4158 LEs, altera EPIS20F780C5, 80 clock cycles	415	-
	SHA-512: 2062 slices, 76 MHz, virtex-II XCV2P7	477	Error detection
	SHA-512: 1584 slices, 41 MHz, virtex-II XCV2P7	524	-
	SHA-1: 539 slices, 130 MHz, virtex-II XCV2P7	408	Error detection
[17]	SHA-1: 422 slices, 66 MHz, virtex-II XCV2P7	422	-
	SHA-512: 2692 slices, 117.15 MHz, virtex-II pro	1’499.58	Fault detection
[20]	SHA-512: 2104 slices, 118.80 MHz, virtex-II pro	1’520.66	-
[18]	SHA-256: 9012 LUT-slices, 112 MHz, virtex 5 XC5VLX330	3’880	Fault detection
	SHA-256: 2255 LUTs, 105.73 MHz, artix-7 XC7A75, 65 clock cycles	277.61	Fault detection
	SHA-256: 1338 LUTs, 97.04 MHz, artix-7 XC7A75, 65 clock cycles	764.37	-
	SHA-256: 2255 LUTs, 168.23 MHz, virtex-7 XC7VH870, 195 clock cycles	441.72	Fault detection
This work	SHA-256: 1350 LUTs, 142.96 MHz, virtex-7 XC7VH870, 195 clock cycles	1’119.04	-

**Table 6:** Test results for fault injection.

Type of Error	Number of Test Vectors	Epochs	Detection Ratio	Correction Ratio
Without error injection	5000	1	100%	N.A.
Stationary (fixed values applied in 1 or 2 clk cycles)	5000	3	100%	100%
Permanent (random values applied in ≥3 clk cycles)	500	3	100%	34.2%

## Data Availability

Not applicable.

## Abbreviations

The following abbreviations are used in this manuscript:

*A, B, …, H*	Working variables
ADDKT	Address of round constant
AES	Advanced Encryption Standard
*B*	SHA-256 simple hardware architecture
$B^{\left(h\right)}$	SHA-256 hybrid redundancy hardware architecture
$B_{k}$	Replica *k* of *B*
$B_{k}^{\left(h\right)}$	Replica *k* of $B^{\left(h\right)}$
BigSigma0	$\Sigma_{0}^{\left(256\right)}$ function
BigSigma1	$\Sigma_{1}^{\left(256\right)}$ function
BG1	Output of $\Sigma_{1}^{\left(256\right)}$ function
BG0	Output of $\Sigma_{0}^{\left(256\right)}$ function
BUFF	Buffer (state) for working variables *A, B, …, H*
CH_Function	*Ch* function
CH	Output of *Ch* function
CBC	Cipher block chaining (CBC)
ECB	Electronic codebook (ECB)
CFB	Cipher feedback (CFB)
OFB	Output feedback (OFB)
CTR	Counter (CTR)
DES	Data Encryption Standard
ECC	Elliptic-curve cryptography
enQ	Enable for storing final hash value
enR	Enable register for initial hash
enSB1	Enable for stage block 1
enSB2	Enable for stage block 2
enSB3	Enable for stage block 3
HOF	New initial hash value
H0R	Initial hash register (it changes if there is more than one message block)
*l*	Latency of *B*
$l^{\left(h\right)}$	Latency of $B^{\left(h\right)}$
*N*	Number of redundancies, where $N\in Z^{+}$
NBUFF	New buffer value
Kbps/LUT	Kilobits per second per LUT
KT	Round constant value
MAJ_Function	*Maj* function
MAJ	Output of *Maj* function
Mbps	Megabits per second
MD5	Message-Digest Algorithm 5
PEA	Processing Element A
PEB	Processing Element B
Reg	Pipeline registers
RSA	Rivest, Shamir y Adleman Algorithms
RST	Reset
selFH	Selector of final hash
selH0	Selector of Initial Hash
selMUX	Selector of Output hash value
selS1	Selector of external/internal message block
SG1	Output of $\sigma_{1}^{\left(256\right)}$ function
SG0	Output of $\sigma_{0}^{\left(256\right)}$ function
SHA	Secure Hash Algorithm
SHA-256	SHA with 256-bit hash
SmallSigma1	$\sigma_{1}^{\left(256\right)}$ function
SmallSigma0	$\sigma_{0}^{\left(256\right)}$ function
TA	Temporary working variable *A*
TE	Temporary working variable *E*
THASH	Temporary intermediate hash value
VHDL	Very high speed integrated circuit hardware description language
WT	Word for each round
1S-HASH	1-stage final temporary hash value
2S-HASH	2-stage final temporary hash value
3S-HASH	3-stage final temporary hash value
